# Parents, Pain, and Over-the-Counter Medicine: Athletes’ Perceived Alternatives to Prescription Opioid Misuse

**DOI:** 10.3390/healthcare11192671

**Published:** 2023-10-02

**Authors:** Rikishi T. Rey, Anne E. Pezalla

**Affiliations:** 1 Department of Communication, Clemson University, Clemson, SC 29634, USA; 2 Department of Psychology, Macalester College, St Paul, MN 55105, USA; apezall1@macalester.edu

**Keywords:** opioids, athlete pain management, youth opioid epidemic, opioid misuse, youth athletes, parent–athlete communication, sport communication

## Abstract

Youth athletes are often prescribed opioids after sustaining sport-related injuries, and because of their age, warrior-like culture in sport, and the desire to perform at the highest level, they are at risk for opioid misuse. Due to the nature of sport, youth athletes are at a greater risk to misuse opioids, and although it does not always predict misuse in adults, it is almost always a precursor among those addicted to opioids in adulthood. This crisis has been classified by the National Center for Health Statistics at the Centers for Disease Control and Prevention as an epidemic, resulting in over a hundred deaths a day and has cost over a billion dollars. To better understand athletes’ experiences and use of opioids, the current study uses in-depth, semi-structured interviews with 35 current athletes and highlights their lived experiences with opioid use. Qualitative, line-by-line coding revealed three main themes: the protective role of parents, the teaching potential of athletes’ own pain, and the easy access to over-the-counter medication and supplements that allow athletes to address their pain or enhance their performance. The results highlight that youth athletes may not misuse opioids to the extent previously predicted by past research and provide insight into the opioid epidemic from a youth sporting perspective.

## 1. Introduction

Prescription opioids have been hypothesized to act as a potential “gateway” to opioid misuse, including recreationally using opioids or sharing one’s prescription opioids with others [1,2]. The National Center for Health Statistics at the Centers for Disease Control and Prevention has called the opioid crisis an epidemic, recording at least 115 deaths each day and resulting in a cost of USD 78.5 billion each year [2,3]. In recent years, it appears that prescription opioid misuse has migrated into the adolescent athletic environment [4]. This shift in context may be because athletes are often at risk for musculoskeletal injuries and commonly play through pain, and that the notion of a warrior mentality has become normalized, especially among high-level athletes [5,6].

Specifically, researchers have found that athletes perceive time off as letting their team down, believing that they will lose their spot as a starter or their role on the team, or jeopardizing their career [7,8]. For example, Cranmer and LaBelle [9] found that high school athletes play through pain in fear of being removed from their sport. Removal from one’s sport can include being cut from the team or being told by a doctor or athletic trainer that they are physically unable to attend a practice or a game. As such, athletes may take medication to help them manage the injury and play through pain [10]. The high school age is of particular interest to this manuscript because of the role parents have in the health decisions of youth athletes as compared with collegiate athletes.

For instance, for youth athletes, it is often forwarded that parents must be able to recognize the signs of an injury and take the necessary steps to ensure the health and safety of their youth athlete [11,12,13]. As Boneau et al. [11] report, parents will have conversations with their children to justify why they are allowing them to play, or continue playing their sport, with a health-related risk or current issue such as an injury. For instance, some parents will allow their children to compete regardless of health status due to the love for the sport itself, whereas other parents perceive that it is the job of the coach or the child to make proper health-related decisions [11]. Indeed, parents’ conversations with their youth athlete, as well as the decisions they make on behalf of their youth athletes (e.g., sitting out of practice and administering medicine), can have significant influences on an athlete’s sporting experience (i.e., playing through pain and injury). Although coaches can significantly influence an athlete’s sporting experiences, due to the age of youth athletes—under the age of 18—the responsibility of prescribed medicines falls onto the athlete’s parents. However, there is a dearth of research that highlights how parents communicate with youth athletes when the athlete is injured or athletes’ perceptions of their parent’s role in their injury management.

As often highlighted, there are differences in athletic ability and performance level at different stages in sports (e.g., high school, collegiate, and professional) [14] as well as different injuries associated with different sports (e.g., contact vs. noncontact). However, regardless of the level or sport, injuries are prevalent in all sports at all levels [15]. For example, when opioid misuse was examined in college student athletes, participants often mentioned that they learned about opioids while in high school from non-evidence-based curricula and that they were simply told not to do drugs—often criticizing the manner in which they were taught—and that it was in high school that they were first prescribed opioids [15]. Notably, high school athletes who use opioids are more susceptible to continued use through college [16], aligning with previous research that indicates that high school athletes have “lifetime opioid use rates of 28% to 46%” [4] (p. 534). Therefore, it is important to address the issue of opioid misuse at its earliest stage to assist athletes in furthering their careers instead of hindering them. However, one of the largest issues regarding this phenomenon is the lack of literature on athlete opioid use specifically from athletes themselves [4].

A possible reason that high school athletes use opioids is the notion that it will make them tough and resilient and improve athletic performance [13]. An important consideration though is that the misuse of opioids among athletes does not lead to positive outcomes. Whereas opioid drugs can initially elicit feelings of pain relief and euphoria seen as a quick fix, the side effects include sedation, confusion, and constipation—and their long-term effects can change heart, lung, and bone function [17]—repercussions that are devastating to athletic performance. Although opioid misuse in adolescence does not always predict opioid misuse in adulthood, it is almost always a precursor among those addicted to opioids in adulthood [18,19,20]. More specifically, although there is literature examining opioid use at the college level (e.g., [21,22]), empirical research on opioid use in high school athletes, from the perspective of the athletes themselves, is still in its infancy.

As part of a larger project, our goals were to learn more about prescription misuse among athletes and then develop a targeted, evidence-based intervention that responds to the unique epidemiology of this public health problem. In order to create such a holistic evidence-based intervention that assists youth athletes in learning about opioids and ways to refuse misuse, we were interested in assessing athletes’ perceptions of their parents’ role in opioid use, why athletes choose to refrain from taking opioids, and if there was anything else they used in place of opioids or to enhance their performances. As such, the current manuscript shares the major findings from the formative interviews conducted with the athletes themselves and answers the following research questions:

RQ1: What are parents’ role in administering prescribed opioids to their youth athlete?

RQ2: Why do youth athletes choose to take or refrain from taking opioid medication?

RQ3: What else, if anything, are athletes taking alongside or instead of opioids?

## 2. Materials and Methods

After receiving approval from IRB, we conducted in-depth semi-structured qualitative interviews, as doing so provided us with the unique ability to understand the context of athletes’ prescription opioid misuse, including the who, what, when, where, and why of the athletes’ decisions [23]. The emic perspectives we gathered from the athletes then provided cognitive and behavioral models used in creating our targeted intervention, as evidence suggests that prevention programs that use content that is germane to its intended audience, that reflects their language, their norms, and their beliefs, will be most effective in enacting the desired behavioral change [24].

### 2.1. Participants

A total of 35 athletes (21 male; 14 female) participated in the interviews, and 24 were used for final coding. Twenty-seven of the athletes played contact sports: football (*n* = 11), soccer (*n* = 7), hockey (*n* = 3), basketball (*n* = 3), wrestling (*n* = 2), and lacrosse (*n* = 1). The remaining athletes played limited contact or no-contact sports: volleyball (*n* = 4), baseball (*n* = 3), and gymnastics (*n* = 1). Participants for this study ranged from 14 to 21 years of age (*M* = 17.8); 60% were Caucasian (*n* = 21), 31% African American (*n* = 11), and 9% Asian (*n* = 3) (see Table 1 for pseudonyms, gender, and sports). Participating athletes were varied in terms of their goals for their respective sports; only one of them had ambitions to play their sport professionally, and the remaining were playing in competitive club leagues or for their high schools. Yet they were all united in that they took their sport seriously, they had played it for most of their lives, and they had done so with an intensity that resulted in a sport-related injury.

### 2.2. Procedures

Participants for the formative research interviews were recruited through ten participating schools across the United States. Advertisements for interview participants were disseminated through Facebook and Instagram requesting participation of athletes ages 14–21 (an age range that included freshman or sophomores in college who could still recall their experiences from high school sports).

All participants engaged in interviews via Zoom that were audio-recorded and transcribed. Participants were asked to share their experiences and assured that there were no right or wrong answers. To protect confidentiality, they were asked to not share full names. Follow-up questions were used to probe more deeply into the athletes’ experiences and to prompt elaboration. Interviews ranged from 28 min to 72 min in length.

Coding the formative interviews occurred in two stages. First, two coders divided the number of transcripts in half and individually identified and categorized key contents of each interview. Data analysis began with line-by-line coding, which involved tagging or selecting meaningful segments of the material that are relevant to the purpose of this study [21]. All tagged segments were provided with a label (e.g., “mom as gatekeeper” or “pain as teacher”). As data were tagged, the segment was compared with all previously coded segments, and if the segment had similar characteristics to previously coded segments, it was assigned that label. If the tagged segment had unique characteristics and could not be coded at any existing label, then it was provided a unique label and definition.

Second, the two coders each coded a subset of the other’s transcripts and met to determine intercoder reliability. Several discrepancies were noted, discussed, and resolved through further refinement of the codebook. The final stage of analysis consisted of pulling together the segmented transcripts into larger themes and recurring ideas. This was conducted both within and across broad groupings to understand athletes’ experiences and identify athletes’ opioid use experiences.

## 3. Results

The following themes emerged from the analysis of athletes’ narratives about managing pain and injuries in their sport: the protective role of parents, the teaching potential of their own pain, and the easier access to over-the-counter medication and supplements that allowed them to address their pain or enhance their performance. These findings are further explained below.

### 3.1. Protective Role of Parents

These athletes often remarked that their parents were important safeguards for them against prescription opioid misuse. Parents’ roles in distributing and managing their athlete’s opioids emerged in four distinct ways: mom as gatekeeper, mom as worrier, dad as fixer, and parents as educators.

#### 3.1.1. Mom as the Gatekeeper

For youth athletes, mothers often enact the role of the gatekeeper. This role meant that the mothers monitored the use of opioids throughout the athlete’s recovery process. For some athletes, this role meant that their mother would keep track of the hours between each dose administered. For example, Brandon, a youth football player said, “She’d say you gotta [sic] wait another three hours, but it’s like well I’m, it hurts real [sic] bad right now.” Mothers often provided other forms of pain medicine in hopes of minimizing the athlete’s exposure to the opioid. Youth football player Jon expressed, “My mom was like, you’re not going to be taking that much oxy [sic] so she switched off between Advil and ibuprofen and then if it was really bad she’d give me the oxycodone right before I went to bed.” Similarly, mothers kept the opioid pill bottles from their children. This restriction meant that she was able to monitor and distribute the pills. Lillie, a volleyball player who initially thought she had tendonitis in her shoulder and tried to play through it and eventually had to have surgery to repair a torn ligament, stated that her “mom keeps the pill boxes.” This type of gatekeeping made it difficult for athletes to misuse opioids. Similar to mothers as gatekeepers, mothers also worried about their athlete’s opioid use, causing athletes to be mindful when taking pills during their recovery process.

#### 3.1.2. Mom as the Worrier

Athletes elaborated that they were mindful of their prescription use because their mothers expressed so much worry regarding the ramifications of improper use. Participants would recall to us the stories they heard from their mothers as they were recovering, stories that cautioned them against misusing their medication with anecdotes about others whose “lives had spiraled” because they themselves had misused. For example, basketball player Jessica explained,

My mom told me a story [while I was recovering from surgery] of a kid who got in a jet ski accident… he got addicted to opioids and his life spiraled after that. He was in jail for a while because he attacked his grandfather when he was high… his life just has spiraled.

Jessica went on to identify that story as the one that forced her to be cognizant of how she managed her own recovery and opioid intake. When mothers shared their worries and concerns with their athletes during the recovery process, it led to athletes taking less than what was prescribed. For example, Rachel stated that her mom, “knows that a lot of people that have gotten [sic] addicted to drugs usually start out after surgeries and pain medicine. She was really worried about that, I kind of was too. So, I didn’t take that many.” Often, the worry that mothers share also causes worry in athletes as it demonstrates the negative consequences of misuse. Notably, mom as the worrier is distinctly different from mom as the gatekeeper as athletes express that the worry communicated to them from mothers was exaggerated concern highlighting the worst-case scenarios of misuse. Although mothers played a predominant role in athletes’ decisions to properly use their prescribed opioids, fathers too had a meaningful role in athletes’ decision-making process.

#### 3.1.3. Dad as the Fixer

Dads had an important role in assisting in the proper use of opioids. Specifically, fathers were seen as the provider of pragmatic guidance on injury prevention and pain management. Dads helped the athletes communicate their pain, acted as the “middle man” to coaches and doctors, and advocated for their athlete in a way that protected them. Football player Brandon explained that his dad was there to communicate to his coach, “[I] went home to my dad like I think I messed up my ankle a little bit, so he called up the coaches and told them what’s going on.” For Stephanie, she turned to him to discuss it, “because he’s a paramedic firefighter… he kind of knows his way around injuries,” explaining that, as a soccer player, she often has knocks and bruises and he helps her determine how to manage pain and injuries. Participants discussed that having their dad there to help them through these situations helped them realize that there was a proper and improper way to manage their injuries, and that opioid misuse was not an option.

#### 3.1.4. Parents as Educators

The athletes elaborated that their parents were there to teach them about the negative consequences of opioid misuse. For some, this education meant that they had conversations even when they were not injured, and for others, it meant that their parents educated them on pain management and how to properly take care of themselves to be able to return to their sport. For Grace, a soccer player, her parents discussed with her the negative health effects of opioids on her liver and the possibility of addiction. This educational information was taken as objective truth for Grace, influencing her decisions to refrain from opioid misuse. Parents also educate their athletes on proper pain management as well. For example, Colleen, a basketball player stated, “She [Mom] talks to me all the time about pain management. She says, if we don’t get this pain under control you’re not gonna [sic] want to play again. You won’t fully heal.” These two examples demonstrate that parents teach their athletes about opioid misuse and proper ways to heal when injured. Similar to how athletes perceived their parents as educating them on proper opioid use and pain management, the athletes also explained that their own pain from injuries taught them a lot, too.

### 3.2. Teaching Potential of Athletes’ Own Pain

For many athletes, they took the absolute minimum of their prescription opioid medication, or nothing at all, to be able to feel and play through pain. To them, being able to play through the pain demonstrated a toughness they thought was necessary. Two main categories emerged from the data set: manageable vs. unmanageable pain and the culture of pain.

#### 3.2.1. Manageable vs. Unmanageable Pain

When injured, many athletes stated that they asked themselves one important question: Can they play through this? For some, the answer was yes; for others, the answer was more complicated, resting not on how much pain they could tolerate but instead how the pain would impact their performance. This nuanced assessment was demonstrated by Lillie, a volleyball player with a shoulder injury. Lillie perceived that she was unable to play when her outside hitting (a positional play in volleyball) was noticeably weaker. “I just could tell,” Lillie told us. “My swing was just not as powerful as it had been. I could feel it wasn’t as strong as before.” Lillie then spoke to us not just to explain how she considered pain in her sport but also how the athletes she knew interpreted pain. She stated: “…pain tolerance with athletes, it’s not necessarily how much pain they can tolerate. It’s a lot to do with when does their performance start to decline?”

Lillie’s exemplary statement illustrates a question to which many participants alluded. What was considered an injury to many would not be for some athletes. For example, a football player, Jimmy elaborated, “I always hear the phrase, you know, you can play through pain, you can play through hurt, you can’t play through an injury” as he explained that playing with four broken fingers made it difficult to catch, impacting his overall performance, but not enough to stop playing. While explaining, he showed in the camera some of his fingers, “Yeah, I would say, like, even a broken finger… this one here [shows finger] it didn’t keep me out of playing… unless you’re like actually out of the sport for X amount of time, you’re not technically injured.” Athletes made their decisions to continue playing through their pain based on an assessment of the pain being manageable or unmanageable in relation to their performance. If they felt that they could continue to perform through the pain, it was not considered a serious injury. This notion correlated with the culture of pain that athletes elaborated on.

#### 3.2.2. Culture of Pain

Athletes also discussed how they like pain. Specifically, participants explained how they want to feel the pain as it is a gauge for how hard they can push themselves. Participants explained that they would rather feel their pain than for it to be masked with opioids, which could lead to more serious injuries. For one football player, Scott, he jokingly stated, “Call me crazy, but I’m one of those people that likes to feel the pain… if I ignore it I know I’m just gonna [sic] hurt myself worse.” Similarly, participants explained that the injuries helped give them the motivation they needed to perform. Dual athlete Addie (swim and basketball) shared, “I’ve liked being aggressive and I like when I got hurt. It just made me even more mad and fueled up to work harder.” Moreover, participants’ reasoning for playing through pain was accompanied by the use and easy access to over-the-counter (OTC) medicine (e.g., acetaminophen and ibuprofen) and other supplements.

### 3.3. Ease of Access to OTCs and Supplements

#### 3.3.1. Anti-Inflammatory Medicine

Participants often explained that they would use more accessible and acceptable uses of pain medicine such as over-the-counter (OTC) anti-inflammatories because, from their knowledge, they are not as addictive or dangerous as opioids. Participants rationalized their decisions by explaining that if they do not need a prescription, then it cannot be that bad for them. For example, Alec, a basketball player, stated, “You don’t need a prescription to take Advil, which to me, says that it’s less dangerous. So, I take Advil.” Participants believe that taking Advil is better than taking opioids even if it means misusing it. Soccer player Rachel explained that she would often misuse anti-inflammatories but felt that it was okay to do so, “I take ‘em [ibuprofen tablets] like candy when I’m in pain. I know I overdo it, but it seems less risky than using opioids.” Often, athletes knew that their teammates would have some form of anti-inflammatory with them and would make comments much like dual athlete (soccer and basketball) Norah’s, “I usually don’t think too much about it because it’s just Advil.” Alongside OTC anti-inflammatories, participants discussed that they would also use other forms of substances.

#### 3.3.2. Protein Powders and Other Supplements

Similar to OTC medication, participants discussed how protein powders and other supplements were considered acceptable. Notably, protein powders and other supplements were not used for pain management but more so for performance enhancement. Some expressed that their teammates had been doing it since high school. Football player Travis elaborated, “My teammates have used [supplements] since like high school.” Travis also explained that he drinks protein shakes not only to continue enhancing his performance but that he gets his protein from a friend’s father. Travis felt that he could trust his friend’s father when he would tell him what he should take since he was in the business. For Jaclyn, a basketball player, she rationalized her use of cannabidiol (CBD) lotions because she perceived them as more natural and uses it for joint and minor pains. Similarly, Grace stated that some alternative remedies that she would use included pain-relieving cream such as Biofreeze. Participants elaborated on the notion that these alternatives assisted them in being able to play at their maximum potential.

## 4. Discussion

The objective of this study was to learn more about prescription misuse among youth athletes in order to develop an evidence-based intervention that responds to this unique public health crisis. Findings suggest that there are three main facets to understand as they pertain to youth athlete opioid misuse: parents’ role in opioid use, athletes’ decision-making process when using or choosing not to use opioids, and if there are other means of pain management. These findings contribute to the growing literature on athletes and opioids [18], and they have practical implications in that they illuminate a currently under-represented protective role of parents in the realm of their children’s prescription opioid usage. Whereas other research has often portrayed parents as “helicopter” authority figures, overly involved in their youths’ nascent athletic careers [25], this study reveals a more hopeful story that many parents authentically want to shield their children from the dangers of prescription opioid misuse and are sincerely trying to help their children manage their pain [11,12,26]. This aligns with previous researchers’ findings that parents focus on their youth athlete’s health, have conversations with them about it, and make decisions for the athletes. More importantly, however, these findings address this phenomenon from the athlete’s perspective—as opposed to the parent’s perspective—and demonstrate that athletes turn to their parents for guidance regarding opioid use. In light of these results regarding parents, prevention and intervention science should incorporate parents as active members into athletes’ pain management plans.

This formative research contributes to an important theme in the research on substance misuse among athletes: This problem involves more than opioids. Notably, athletes communicated that although they were less inclined to misuse opioids, their use (and sometimes misuse) of OTC medication was acceptable. This is an interesting finding as previous literature has noted that OTC medications are often all that is needed for athletes to manage their pain [13]. However, the study inadvertently did not address the ramifications of OTC misuse in lieu of opioid misuse. The danger of this logic should be further explored to a greater extent as it illustrates athletes’ decision-making and rationale behind how and why they choose to play through pain. Future research should investigate these options in more detail to examine the other factors or experiences that accompany the distinct developmental pathways of OTC misuse and opioid misuse.

Finally, another implication and contribution of this research is that many athletes alluded to the notion that the rigidity of sport instills in them the understanding that they must follow the rules to succeed. Notably, the idea that they must follow the rules then leads to addictive personalities that they are mindfully aware of. Such characteristics may be what influences their decision-making processes in their sport involvement. As such, future research should explore the relationship between athletes and coaches and how rule-abiding socialization may contribute to pain management behaviors. Practically, this study highlights the findings presented by McLean [15] and the need for an evidence-based curriculum to educate athletes on opioids and ways they can communicatively refuse opioid misuse (e.g., sharing), highlight the negative effects of a warrior culture mentality, as well as address the ramifications of OTC medicine misuse.

Notably, this study is not without its limitations. First, our sample was small in size, and future research should expand to larger samples. Second, our sample was limited in its diversity with opioids: Although recruited participants were only those who had experienced a sport-related injury, many who participated were those who were still playing and who had specifically chosen to properly use opioids or not use them at all. Furthermore, our participating athletes were not playing professionally and thus may have had fewer pressures to misuse opioids to excel in their sport. Future research should investigate professional athletes, athletes who may have quit their sport due to opioid addiction, and those who used opioids to manage pain and avoid surgical interventions. Findings would result in understanding the decisions that lead to opioid misuse and may assist in combating misuse.

## 5. Conclusions

In conclusion, this research provides meaningful and insightful information regarding youth athletes’ opioid use and misuse. Specifically, it draws attention to the important role parents provide in assisting athletes with proper opioid use, highlights the interesting context sports provide as it pushes a culture of feeling pain instead of masking it, and addresses the issue of OTC medications that youth athletes may not perceive as harmful. More notably, the current study presents a novel conclusion that athletics may provide athletes with a specific context that encourages athletes to properly use pain medication and that the presence of parents in athletes’ recovery journey may also assist in the proper use of opioids. Although athletics is not preventative or a solution to the youth opioid epidemic, it does provide more understanding to our knowledge of youth athletes’ opioid use.

## Figures and Tables

**Table 1:** Participants.

Pseudonym	Gender	Sport
Brandon	Male	Football
Zac	Male	Wrestling
Lillie	Female	Volleyball
Henry	Male	Football
Stephanie	Female	Soccer
Keith	Male	Football
Jimmy	Male	Football
Stacey	Female	Soccer
Addie	Female	Swim & Basketball
Casey	Female	Volleyball
Danny	Male	Football
Colleen	Female	Basketball
Jessica	Female	Basketball
Mark	Male	Baseball
Alec	Male	Basketball
Rachel	Female	Soccer
Scott	Male	Football
Travis	Male	Football
Greg	Male	Wrestling
Tess	Female	Volleyball
Jaclyn	Female	Basketball
Jason	Male	Tennis
Norah	Female	Soccer & Basketball
Grace	Female	Soccer

Participant interviews used for this study (*n* = 24).

## Data Availability

Please contact Rikishi T. Rey at rrey@clemson.edu for access to the analyzed data.
